# Ethanol Negatively Regulates Hepatic Differentiation of hESC by Inhibition of the MAPK/ERK Signaling Pathway *In Vitro*

**DOI:** 10.1371/journal.pone.0112698

**Published:** 2014-11-13

**Authors:** Wei Gao, Ping Zhou, Xiaocui Ma, Benjamin Tschudy-Seney, Jiamei Chen, Nataly L. Magner, Alexander Revzin, Jan A. Nolta, Mark A. Zern, Yuyou Duan

**Affiliations:** 1 Department of Biomedical Engineering, College of Biology, Hunan University, Changsha, Hunan, China; 2 Department of Internal Medicine, University of California Davis Medical Center, Sacramento, California, United States of America; 3 Institute for Regenerative Cures, University of California Davis Medical Center, Sacramento, California, United States of America; 4 Department of Biomedical Engineering, University of California Davis, Davis, California, United States of America; 5 Institute of Liver Diseases, Shuguang Hospital, Shanghai University of Traditional Chinese Medicine, Shanghai, China; National Cancer Institute, United States of America

## Abstract

**Background:**

Alcohol insult triggers complex events in the liver, promoting fibrogenic/inflammatory signals and in more advanced cases, aberrant matrix deposition. It is well accepted that the regenerative capacity of the adult liver is impaired during alcohol injury. The liver progenitor/stem cells have been shown to play an important role in liver regeneration -in response to various chronic injuries; however, the effects of alcohol on stem cell differentiation in the liver are not well understood.

**Methods:**

We employed hepatic progenitor cells derived from hESCs to study the impact of ethanol on hepatocyte differentiation by exposure of these progenitor cells to ethanol during hepatocyte differentiation.

**Results:**

We found that ethanol negatively regulated hepatic differentiation of hESC-derived hepatic progenitor cells in a dose-dependent manner. There was also a moderate cell cycle arrest at G1/S checkpoint in the ethanol treated cells, which is associated with a reduced level of cyclin D1 in these cells. Ethanol treatment specifically inhibited the activation of the ERK but not JNK nor the p38 MAP signaling pathway. At the same time, the WNT signaling pathway was also reduced in the cells exposed to ethanol. Upon evaluating the effects of the inhibitors of these two signaling pathways, we determined that the Erk inhibitor replicated the effects of ethanol on the hepatocyte differentiation and attenuated the WNT/β-catenin signaling, however, inhibitors of WNT only partially replicated the effects of ethanol on the hepatocyte differentiation.

**Conclusion:**

Our results demonstrated that ethanol negatively regulated hepatic differentiation of hESC-derived hepatic progenitors through inhibiting the MAPK/ERK signaling pathway, and subsequently attenuating the WNT signaling pathway. Thus, our finding provides a novel insight into the mechanism by which alcohol regulates cell fate selection of hESC-derived hepatic progenitor cells, and the identified pathways may provide therapeutic targets aimed at promoting liver repair and regeneration during alcoholic injury.

## Introduction

The liver is the major location for the metabolism of ethanol, and alcoholic hepatitis and other forms of alcoholic liver disease (ALD) are major complications of chronic excessive ethanol intake [1], [2]. At an early stage in the course of alcohol-induced liver injury, damaged hepatocytes can be replaced by the proliferation of adult hepatocytes. However, with the course of more progressive and chronic injury, hepatocyte proliferation becomes less successful in the re-establishment of an adequate hepatocyte mass for the restoration of liver function. At that stage, the differentiation of hepatic stem/progenitor cells becomes critical in hepatocyte regeneration and in the other elements of the repair process, including fibrogenesis. Although the types and nomenclature of liver stem/progenitor cells are in some dispute, and differ in rodents and humans, there is some consensus that they evolve from bipotent stem cells that resides within the Canal of the Hering between the hepatocyte plate and bile duct. These liver stem/progenitor cells are shown to give rise to both hepatocytes and cholangiocytes in response to various chronic injuries [3], [4].

The effects of alcohol injury of adult liver cells have been studied extensively. Alcohol injures hepatocytes and activates stellate cells as well as Kupffer cells, leading to a loss of hepatic function, aberrant deposition of ECM proteins and production of inflammatory and profibrogenic signals [5], [6], [7], [8]. However, relatively little is known about the human liver stem cell response to this toxicant [9]. While the isolation of human hepatic progenitor cells has been reported in the literature [10], [11], [12], the scarcity of human livers and small numbers of progenitor/stem cells in the liver make it impractical to conduct mechanistic studies of alcoholic injury on liver progenitor/stem cells *in vivo*. Thus, other approaches must be made to employ an appropriate *in vitro* model to evaluate the impact of alcohol on liver progenitor/stem cells. Hepatic derivatives from human embryonic stem cells (hESCs) provide promising resources to acquire knowledge of the cellular and molecular bases underlying human liver development and pathological conditions. A recent report evaluated ethanol treatment during the middle and late stages of hepatic differentiation from hESCs, thus mimicking how alcohol may cause liver damage in vivo using an in vitro model employing hESCs [13]. We employed hESCs to progressively differentiate them into definitive endoderm (DE) cells, then hepatic progenitor cells, and finally hepatocytes [14], [15], [16], [17], [18], [19], [20], [21]. Thus, hESC-derived hepatic progenitor cells after the DE stage can be used as an alternative to bioptent liver progenitor/stem cells, In the present study, we treated the hESC-derived hepatic progenitor cells with ethanol immediately after the DE stage, and evaluated the effects of ethanol on early hepatic differentiation with an attempt to mimic how alcohol modulates the differentiation of hepatic progenitor cells in vivo using this in vitro model employing hESC. We further investigated the mechanism by which ethanol modulated the hepatic differentiation from these hepatic progenitor cells.

## Materials and Methods

### Cell culture

The human embryonic stem cells (hESC), H9 line, was purchased from WiCell Research Institute (Madison, WI), and maintained and expanded on mouse embryonic fibroblasts (MEFs) as instructed by the provider.

### Differentiation of hESC towards hepatocytes

Hepatocyte differentiation started from the induction of definitive endoderm (DE) from hESCs using previously described protocols [21]. Briefly, hESCs was induced to DE in RPMI medium (Invitrogen) with 100 ng/ml activin A (R & D Systems Inc.) without serum for 2 days and then the medium was changed to RPMI medium with 100 ng/ml activin A, 0.5 mM sodium butyrate and 1×B27 supplement (Invitrogen) for 6 days. DE cells were then split and re-seeded on collagen I-coated 6-well plates (BD) in IMDM media (Invitrogen) supplemented with 20% FBS (Invitrogen), FGF-4 (20 ng/ml), HGF (20 ng/ml), BMP2 (10 ng/ml), BMP4 (10 ng/ml) (R and D), 0.3 mM 1-thioglycerol (Sigma), 0.5% DMSO (Sigma), 100 nM dexamethasone (Sigma) and 0.126 U/ml human insulin (Hospira, Inc) for 2 weeks. Then the cells were further differentiated and maturated in hepatocyte culture medium supplemented with SingleQuots (Lonza), 0.5% DMSO, 100 nM dexamethasone, 20 ng/ml FGF4, 20 ng/ml HGF, and 50 ng/ml oncostatin M (R & D systems) until use.

### Treatment of differentiated cells with ethanol

Ethanol was used to treat the differentiating cells daily at day 2 after the DE cells were re-seeded on collagen I-coated plates for differentiation to hepatocytes at three concentrations of 25 mM, 50 mM, and 100 mM until use.

### Immunohistochemistry

Differentiated cells were fixed with 4% paraformaldehyde and analyzed by immunohistochemistry as described previously by us [21]. Antibodies used are listed in Table S1.

### MTT viability assay

MTT (3-(4,5-dimethyl-2-thiazolyl)-2,5-diphenyltetrazolium bromide) (Sigma-Aldrich) was added to the cell cultures at the final concentration of 0.5 mg/ml. Culture media was aspirated 3–4 h later. The products of MTT were dissolved in DMSO and measured for absorbance at 570 nm.

### Quantitative RT-PCR (qRT-PCR)

Differentiated cells were harvested at different time points after differentiation, and total RNA isolation, cDNA generation and quantitative real-time PCR were carried out as previously described [21]. Primers or primers/probes used are listed in Table S2.

### Western Blot Analysis

Differentiated cells were lysed in RIPA buffer with proteinase inhibitor cocktail and 5 mM EDTA (Thermo Fisher Scientific) at different time points after differentiation. 30–50 µg of total proteins or 5–10 µg of nuclear protein was used for immunoblot analysis as previously described [22]. Antibodies used are listed in Table S1.

### Enzyme-Linked Immunosorbent Assay analysis

The enzyme-linked immunosorbent assay (ELISA) was used to determine human ALB values secreted into the medium by differentiated cells. ELISA analysis was performed as previously described [21].

### Small Molecule Inhibitors

U0126 (Erk inhibitor, EMD4Biosciences) and IWR-1-endo (WNT1 inhibitor, Tocris), were dissolved in DMSO and used at the concentration of 10 and 0.2 µM, respectively from day 2 after differentiation until use.

### Rescue of impaired hepatocyte differentiation

Rescue of impaired hepatocyte differentiation was performed by supplementing high dose FGF-4 or HGF at the range of 40 ng/ml to 100 ng/ml in the presence of U0126 at the concentration of 10 µM.

### Flow cytometry for cell cycle analysis

The differentiated cells treated with or without ethanol were harvested, permeabilized, fixed in ethanol. Prior to the staining, the cells were treated with RNase A to remove RNAs from the cells. Then propidium iodide staining and flow cytometry was performed for cell cycle analysis as described [23], [24].

### PCR Array assay

Differentiated cells were harvested at different time points after differentiation, and total RNA isolation, cDNAs were generated using RT2 First Strand Kit (Qiagen), Signal Transduction PathwayFinder PCR Array (PAHS-014Z, Sabiosciences) was performed using RT2 SYBR Green qPCR Mastermix (Sabiosciences), and data was analyzed as instructed by the provider. The 84 genes used in PCR Array are listed in Figure S1.

### Statistics

All data were summarized as means ± SEM from at least three independent measurements. An unpaired Student t test was used to analyze the data. p<0.05 was considered statistically significant.

## Results

### Ethanol impaired hepatocyte differentiation from hESC-derived hepatic progenitor cells

**Figure 1:**
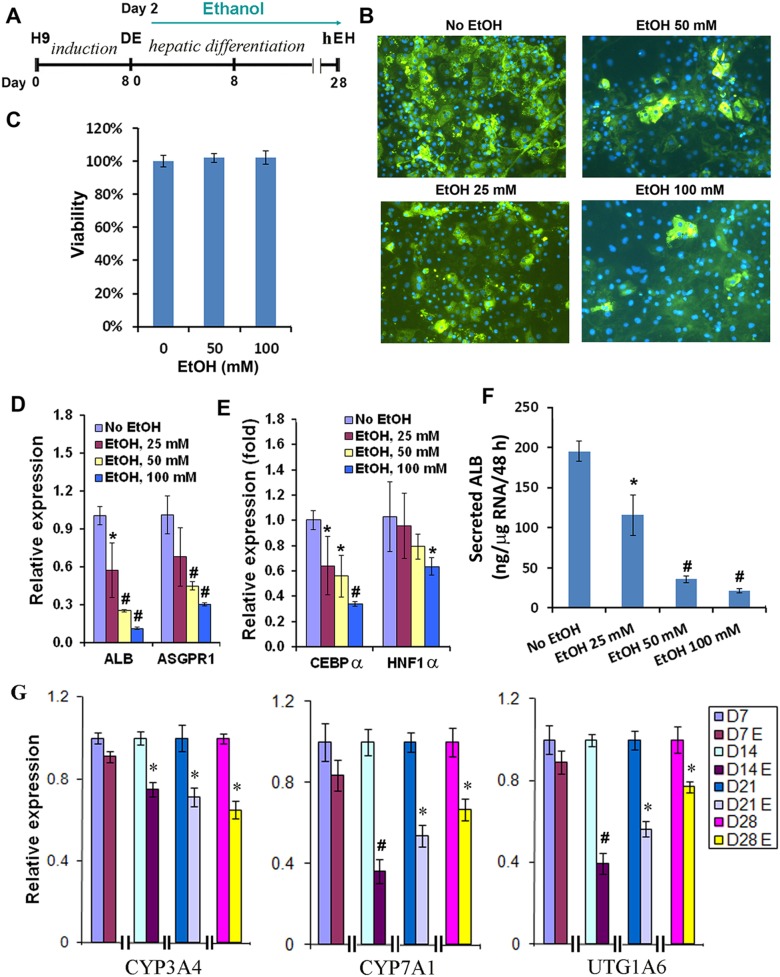
Effects of ethanol on the differentiation of hESC towards hepatocytes. (A) Schematic illustration of the differentiation protocol and the time frame of ethanol treatment. (B) Immunostaining of albumin in the hESC-derived hepatocytes (hEH) at day 24 exposed to the indicated doses of ethanol (x100). (C) Cell viability was assessed at day 24 by MTT assay. (D, E) qPCR was performed to determine the expression of liver genes, ALB and ASGPR1, C/EBPα, and HNF1α at day 24 exposed to the indicated doses of ethanol. (F) Secreted ALB levels in the culture media by hEH were assessed by ELISA at day 24 with or without ethanol treatment. (G) Dynamic expression changes of metabolizing enzymes, CYP3A4, CYP7A1, and UTG1A6 in hEH were evaluated by qPCR at different time points after differentiation in the presence or absence of ethanol at 100 mM. (*p<0.05 vs. no EtOH; #p<0.005 vs. no EtOH).

We employed our previously developed protocol to progressively differentiate hESCs to definitive endoderm (DE), hepatic progenitor cells and hESC-derived hepatocyte (hEHs) (Figure 1A) [21]. The differentiating cells after the DE stage were treated daily with ethanol at three final concentrations, 25 mM, 50 mM, 100 mM. Cells not exposed to ethanol were used as a control. We determined that exposure to ethanol resulted in a dose-dependent decrease in the number of albumin (ALB) positive cells in the cultures, determined by immunohistochemistry (Figure 1B). The reduced number of ALB expressing cells was not due to the toxic effect of ethanol on these cells because ethanol, even at 100 mM, did not significantly alter the viability of the cells as determined by the MTT assay (Figure 1C). Consistent with the decreased number of ALB positive cells in the ethanol-treated cultures, the expression level of albumin and another marker of a mature hepatic phenotype, asialoglycoprotein receptor 1 (ASGPR1), as determined by qRT-PCR, was also markedly reduced in the cells treated with 50 or 100 mM ethanol (Figure 1D). Liver specific transcription factor C/EBPα has been shown to play an important role in hepatocyte differentiation, and it was significantly reduced by ethanol at all tested concentrations as assessed by qRT-PCR, whereas the expression of HNF1α was markedly down-regulated by the ethanol treatment at 100 mM (Figure 1E). A functional assay also showed that the amount of secreted albumin was markedly reduced by ethanol in a dose-dependent manner (Figure 1F). Metabolism and detoxification are important function of hepatocytes, in which cytochrome P450 (CYP) and UGT play key roles. We evaluated several important phase I and II metabolizing enzymes in the cells treated with ethanol at 100 mM, and found that the expression of CYP3A4, CYP7A1, and UGT1A6, was significantly reduced by ethanol (Figure 1G). Therefore, our results demonstrated that hepatocyte differentiation from hESC-derived hepatic progenitor cells was markedly impaired by exposure to ethanol.

### Ethanol induced a moderate growth arrest in hESC-derived hepatic progenitor cells

**Figure 2:**
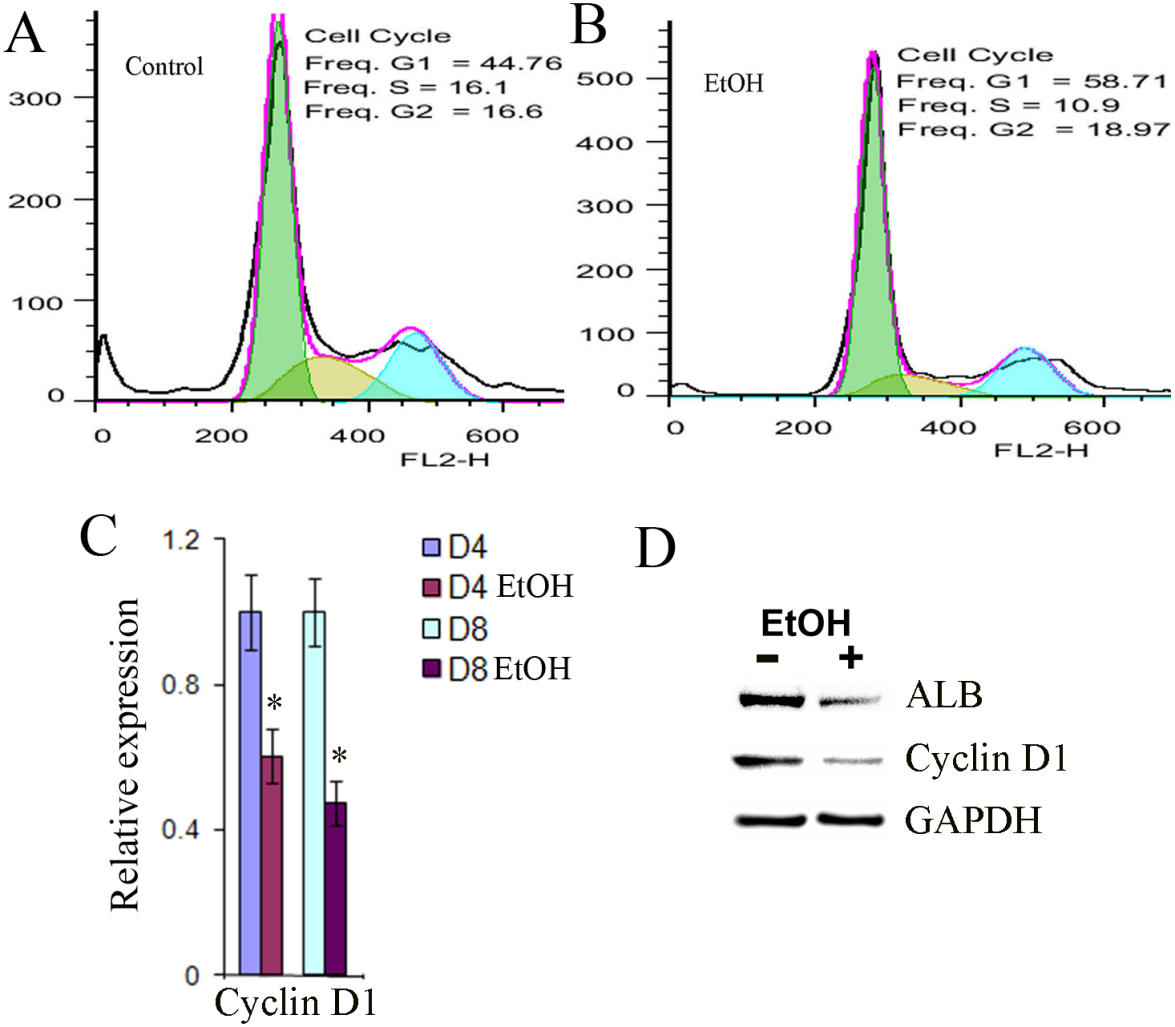
Effects of ethanol on cell proliferation. (A, B) Flow cytometry to assess the amount of DNA in the G1, S, and G2 phases during the cell cycle at day 8 after differentiation in the presence or absence of ethanol at 100 mM. (C) qPCR was performed to evaluate the expression of cyclin D1 at days 4 and 8 after differentiation in the presence or absence of ethanol at 100 mM (*p<0.05 vs. no EtOH). (D) Western blot analysis was used to determine protein expression of albumin (ALB), and cyclin D1 at day 8 after differentiation in the presence or absence of ethanol at 100 mM. GAPGH was used as housekeeping gene control.

To assess the effect of ethanol on the proliferation of the hESC-derived hepatic progenitor cells, these progenitor cells were exposed to ethanol for 8 days, and their cell cycle distribution was determined by flow cytometry. Ethanol treatment increased the population in G1 phase and reduced the population in S phase (Figure 2B) compared to the control cells (Figure 2A), suggesting that a cell cycle arrest at G1/S phase was induced in ethanol-exposed cells. Since G1/S transition is controlled by CDK4/6-cyclin D complexes, we next assessed the level of cyclin D1 in the progenitor cells with or without ethanol treatment by qPCR and Western blot. Consistent with the G1/S phase arrest, the level of cyclin D in ethanol exposed cells was reduced (Figure 2C and D). Thus, ethanol induced a moderate growth arrest at the G/S phase in hESC-derived hepatic progenitor cells associated with a decreased cyclin D 1 expression.

### Several alcohol metabolizing enzymes were up-regulated during hepatocyte differentiation

**Figure 3:**
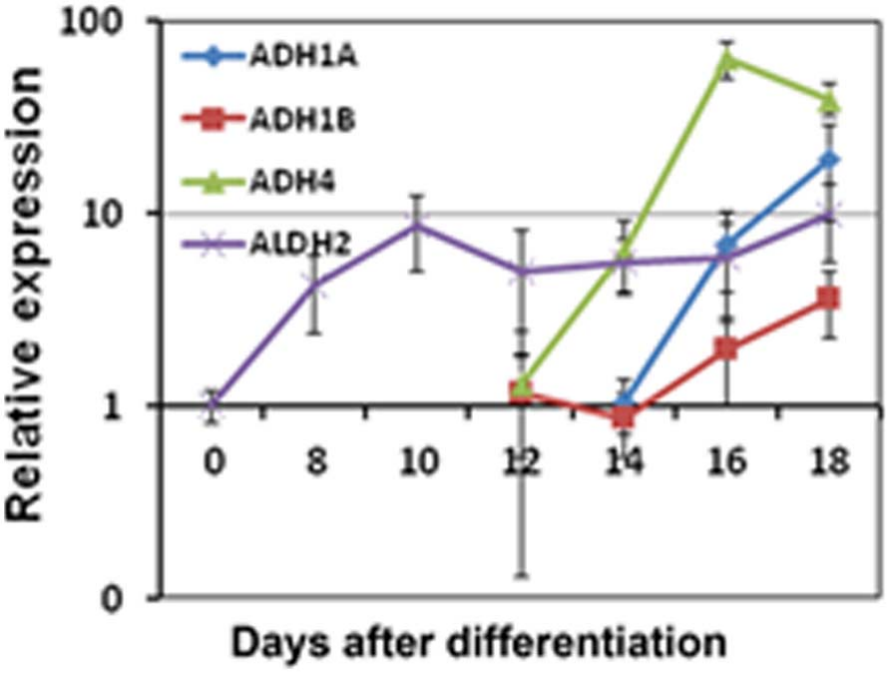
Expression of ethanol metabolizing genes during hepatocyte differentiation of hESC. qRT-PCR was employed to evaluate the dynamic expression of alcohol dehydrogenases (ADHs), ADH1A, ADH1B and ADH4, and by aldehyde dehydrogenases (ALDH), ALDH2 during the hepatocyte differentiation from ESC-derived hepatic progenitor cells.

Ethanol is metabolized first to acetaldehyde primarily by alcohol dehydrogenases (ADHs) and the cytochrome p450 family protein CYP2E1 [25]. Acetaldehyde is toxic to cells by inducing oxidative stress in the liver, and can be further converted into acetate by aldehyde dehydrogenases (ALDH) [25]. We found that the expression of ADH1A, ADH1B and ADH4 were substantially induced during the hepatocyte differentiation from hESC-derived hepatic progenitor cells (Figure 3). The expression of ALDH2 was induced very early at DE stage and the elevated level was maintained during the remainder of hepatocyte differentiation (Figure 3). Thus, our data suggest that hepatocyte progenitor cells derived from hESCs have the capacity to metabolize ethanol.

### Ethanol inhibited the ERK MAPK signaling pathway

**Figure 4:**
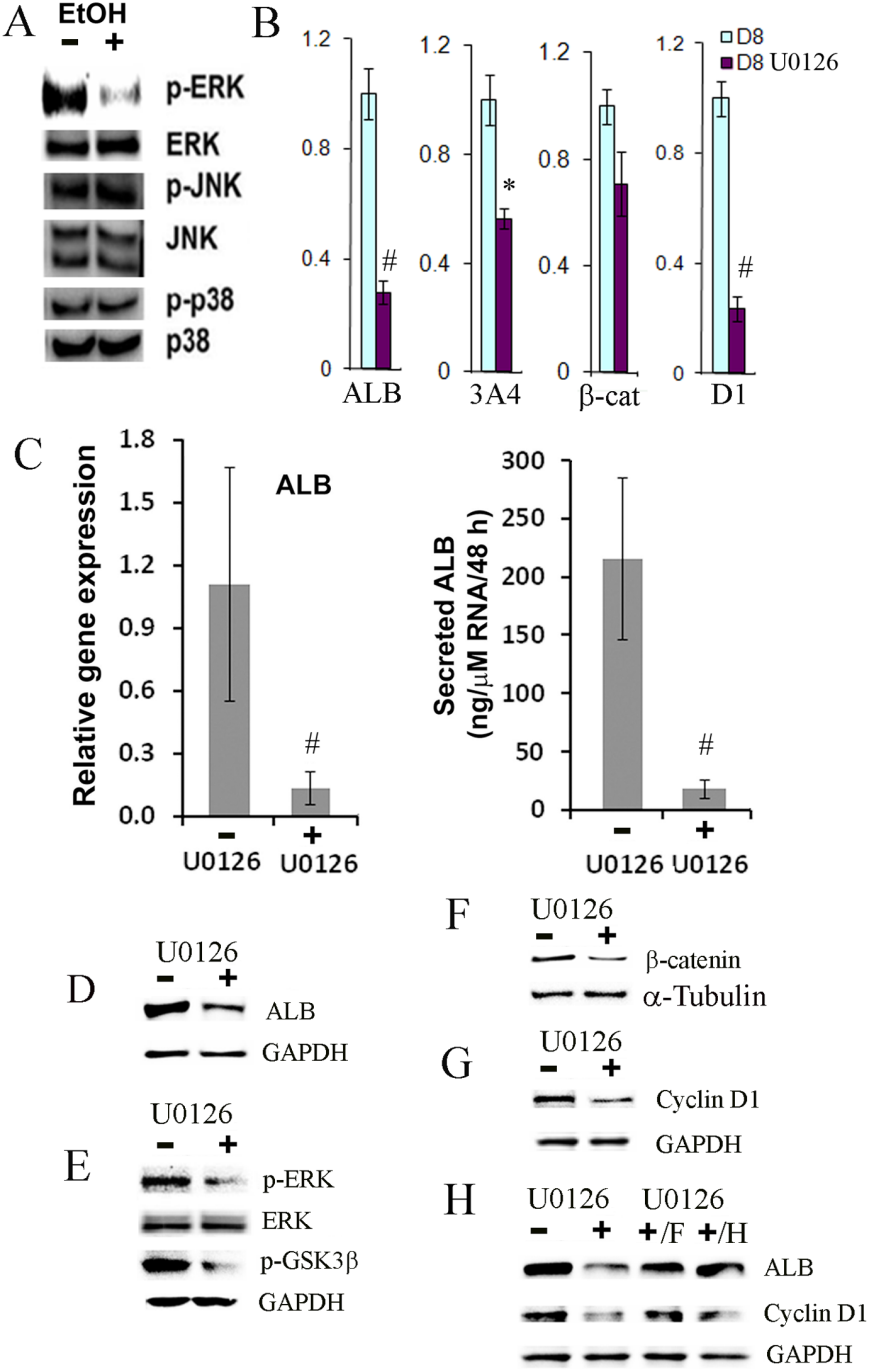
Effects of U0126, Erk inhibitor, on the differentiation of hESC towards hepatocytes. (A) Western blot analysis was employed to determine the effect of ethanol on the MAPK signaling pathway at day 8 after differentiation in the presence or absence of ethanol at 100 mM. (B) qPCR was used to determine the expression of albumin (ALB), CYP3A4 (3A4), β-catenin (β-cat), and cyclin D1 (D1) at day 8 after differentiation in the presence or absence of U0126 at 10 µM (*p<0.05 vs. no inhibitor; #p<0.005 vs. no inhibitor). (C) qPCR and ELISA were employed to evaluate the relative expression levels of albumin (ALB) and secreted ALB into the medium in the same samples in the presence or absence of U0126 at 10 µM. (p<0.05 vs. no inhibitor). (D–G) Western blot analysis was performed to determine protein expression of albumin (ALB), phosphorylated Erk (p-ERK), phosphorylated GSK-3β at the Ser9 residue (p-GSK3β), and cyclin D1 cytoplasm, and β-catenin in nucleus at day 8 after differentiation in the presence or absence of U0126 at 10 µM. GAPDH and α-Tubulin were control housekeeping genes in cytoplasm and nucleus. (H) Western blot analysis was used to evaluate the expression of albumin (ALB), and cyclin D1 by supplementing high dose FGF-4 (F) or HGF (H) at day 8 after differentiation in the presence or absence of U0126 at 10 µM.

The PI3 K/AKT and MAPK signaling pathways have been shown to be involved in early liver development and hepatic differentiation [26], [27], [28]. In order to investigate how ethanol alters hepatic differentiation at the molecular level, we assessed the effect of ethanol on the activation of the PI3 K/AKT and MAPK pathways. We found that exposure to ethanol resulted in reduced activation/phosphorylation of ERK but not JNK and p38 in the MAPK pathway, as determined by Western blot (Figure 4A). The activation/phosphorylation of AKT was only transiently reduced in the presence of ethanol (data not shown).

In order to further investigate whether the ERK signaling pathway regulates hepatocyte differentiation, cells were treated with an inhibitor of MEK1/2, U0126, at 5 or 10 uM from day 2 to day 10 during hepatocyte differentiation of hESC-derived progenitor cells. It is known that activated MEK1/2 specifically catalyzes the phosphorylation of ERK1/2, and thus U0126 can block the activation of ERK. The low concentration of U0126 was chosen to mimic the partial inhibition of ERK activation elicited by ethanol. Inhibition of the ERK signaling pathway markedly decreased the expression of ALB, CYP3A4, as determined by qPCR and Western blot (Figure 4B and D). Furthermore, a functional assay showed that albumin secretion was also markedly reduced by the inhibition of the ERK signaling pathway (Figure 4E). We confirmed that the phosphorylated Erk (active Erk) was indeed reduced by U0126 (Figure 4C). Inhibition of ERK activation by U0126 also reduced the level of phosphorylated GSK-3β and nuclear β-catenin (Figure 4B, E and F), suggesting a reduced activation of Wnt signaling pathway when ERK activation was inhibited. Furthermore, the expression of cyclin D1 was significantly down-regulated in the presence of U0126, as determined by both qPCR and Western blot (Figure 4B and G). Therefore, our results suggested that ethanol compromised hepatocyte differentiation by inhibition of ERK activation.

FGF4 and HGF have been shown to promote hepatocyte differentiation via the MAPK/ERK pathway (14, 34). Hence in order to investigate whether impaired hepatocyte differentiation can be rescued in the presence of the ERK inhibitor, different doses FGF4 and HGF at 20, 40, 60, 80 and 100 ng/ml were supplemented in the differentiation medium in the presence of U0126. Either FGF4 or HGF at 60 ng/ml or higher restored the expression of ALB and cyclin D1 in the cells even in the presence of U0126 (Figure 4H), indicating that impaired hepatocyte differentiation and proliferation was partially rescued.

### Ethanol reduced the WNT signaling pathway in hESC-derived hepatic progenitor cells

**Figure 5:**
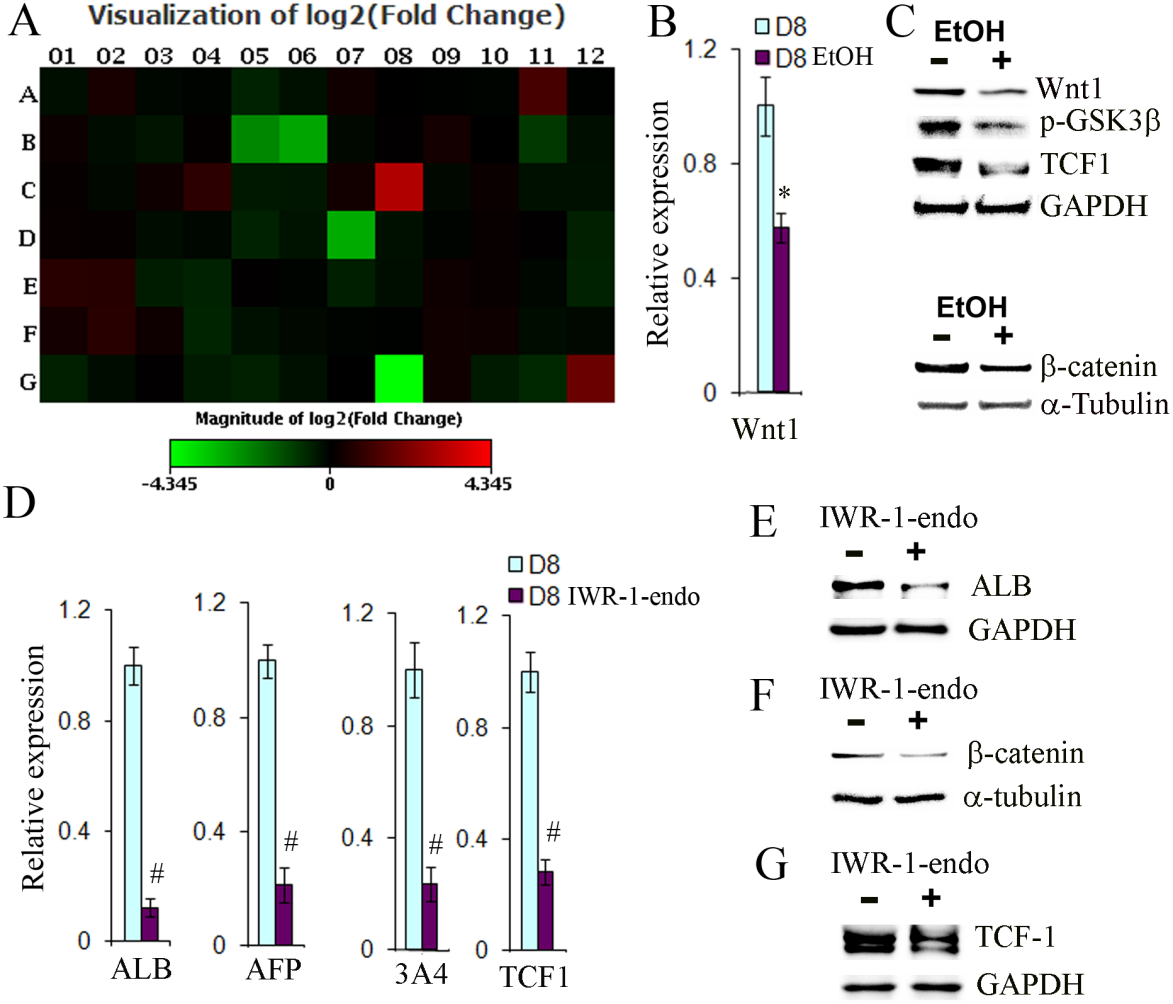
Effects of IWR-1-endo, a WNT1 inhibitor, on the differentiation of hESC towards hepatocytes. (A) The entire view of the expression changes of 84 genes representing 10 pathways by PCR Array analysis at day 10 after differentiation in the presence or absence of ethanol at 100 mM. The names of the 84 genes and their expression fold changes are listed in Figure S1. (B) qPCR was performed to evaluate the expression of Wnt1 at 8 after differentiation in the presence or absence of ethanol at 100 mM (p<0.05 vs. no inhibitor). (C) Western blot analysis was used to determine protein expression of Wnt1, phosphorylated GSK-3β at the Ser9 residue, and TCF1 in cytoplasm, and β-catenin in nucleus at day 8 after differentiation in the presence or absence of ethanol at 100 mM. GAPGH and α-Tubulin were used to as housekeeping gene controls in the cytoplasm and nucleus respectively. (D) qPCR was used to measure the expression of albumin (ALB), alpha fetoprotein (AFP), CYP3A4 (3A4), and TCF1 at day 8 after differentiation in the presence or absence of IWR-1-endo at 0.2 µM (#p<0.005 vs. no inhibitor). (E–G) Western blot analysis was employed to determine protein expression of albumin (ALB), and TCF1 in cytoplasm, and β-catenin in nucleus at day 8 after differentiation in the presence or absence of IWR-1-endo, at 0.2 µM. GAPDH and α-Tubulin as control housekeeping genes in cytoplasm and nucleus.

In order to investigate whether specific signaling pathways are involved in hepatic differentiation in the presence of ethanol, the Signal Transduction PathwayFinder PCR Array containing 84 genes representing 10 pathways was employed to assess the effects of ethanol treatment on the progenitor cells. Four genes including Wnt1 were found to be down-regulated by ethanol (Figure 5A, Figure S1). Since the Wnt signaling pathway has been associated with hepatocyte and cholangiocyte differentiation [29], [30], [31], we sought to further determine whether this pathway was indeed affected by ethanol. The reduced expression of Wnt 1 in the ethanol- treated progenitor cells was confirmed by qPCR and Western blots (Figure 5B and C). Phosphorylation of GSK-3β, β-catenin nuclear translocation, and TCF1 up-regulation have been shown to be involved in the Wnt pathways [32], [33], [34]. The phosphorylation of GSK-3β is essential for de-phosphorylation of β-catenin and release of β-catenin from the destructive complex to translocate into the nucleus [32]. To further assess whether these downstream Wnt signaling components are altered by ethanol, the level of phosphorylated GSK-3β, TCF1 and nuclear β-catenin in the progenitor cells treated with or without ethanol was determined by Western blot analysis. We found that ethanol markedly reduced the level of phosphorylated GSK-3β as well as the level of TCF1 and nuclear β-catenin (Figure 5C). Thus, our data demonstrated that the WNT pathway was attenuated during early hepatic differentiation in the presence of ethanol.

In order to further investigate whether Wnt signaling regulates hepatocyte differentiation from hESC-derived hepatic progenitor cells, the Wnt inhibitor, IWR-1-endo, was used to treat cells at the final concentration of 0.2 µM from day 2 to day 8 during the differentiation. The expression of ALB, AFP, and CYP3A4 was dramatically decreased by the inhibitor, as determined by qRT-PCR and Western blot (Figure 5D and E). We determined that IWR-1-endo indeed reduced the amount of β-catenin in the nucleus (Figure 5F), as well as TCF1, a β-catenin target gene (Figure 5F). Our data suggest that Wnt signaling may be required for hepatocyte differentiation, and may also be affected by ethanol treatment.

## Discussion

In liver development, expression of mesenchymal WNT and fibroblast growth factor 4 (FGF4) signaling in the foregut enables liver and pancreas induction [26], [35], [36]; afterwards, FGF from the cardiac mesoderm and BMP from septum transversum mesenchymal cells coordinately induce liver differentiation and suppress the pancreatic differentiation [25], [26], [37], [38], [39] in the ventral foregut. MAPK is activated in response to FGF in the lateral hepatic progenitors [26], [40]. The newly specified hepatic cells in embryos are hepatoblasts, which express liver specific genes and are bipotent, and these cells further differentiate into hepatocytes and cholangiocytes [26], [41]. Recent studies suggest an important role for FGF/extracelluar signal-regualted kinase (Erk) signaling in promoting the transition from a naïve state to a primed state in pluripotent stem cells [42], [43], [44], [45], [46]. FGF4 is the major stimulus activating Erk in embryonic stem cells as FGF4-stimulated activation of Erk1/2 is an autoinductive stimulus for naïve ES cells to exit self-renewal, and the Erk cascade directs transition to a state that is responsible to inductive cues for germ layer segregation [47]. Other studies have shown that FGF4 signaling directs endoderm lineages [42]. In addition, MAPK (JNK, p38, and Erk) signaling pathways have been shown to play an important role in mediating stem cell proliferation and differentiation [48], [49], [50]. These pathways constitute a large kinase network that regulates a variety of physiological process, including cell growth, differentiation, and apoptotic cell death, in response to a diverse set of stimuli. Moreover, recent studies have indicated that FGF4 and HGF promote the differentiation of mouse bone marrow mesenchymal stem cells towards hepatocytes via the MAPK (p-38 and Erk) pathway [28].

In our differentiation conditions, FGF4, HGF, BMP2 and BMP4 were employed to induce early hepatic differentiation [21], mimicking in vivo liver development. Importantly, we determined that ethanol inhibited hepatocyte differentiation form hESC-derived hepatic progenitor cells and Erk activation was attenuated by the treatment with ethanol, indicating that the activation of Erk appears to be a critical pathway which directs the hepatic progenitors to differentiate towards hepatocytes under our differentiation condition (Figure 4A). We further revealed that inhibition of Erk activity by ethanol resulted in the inhibition of the phosphorylation of GSK-3β at the Ser9 residue, which in turn decreased the translocation of β-catenin into the nucleus. Consistent with the reduced WNT signaling, the expression of β-catenin target genes, TCF1 and cyclin D1, was also down-regulated in ethanol exposed cells. The reduced level of cyclin D in these cells led to a moderate cell cycle arrest at G1/S checkpoint. This pathway was confirmed by employing the Erk inhibitor, U0126and the level of p-Erk and p-GSK-3β, nuclear β-catenin, and cyclin D1 was significantly decreased in the presence of U0126 (Figure 4). Similar to ethanol treatment, the Erk inhibitor also markedly reduced the expression of many liver specific genes and the secretion of ALB (Figure 4). The expression of albumin and cyclin D1 in cells exposed to U0126 could be partially rescued by excess doses of FGF4 or HGF (Figure 4H). We have shown previously that FGF4 and HGF potently activates ERK, JNK and p38 MAPK pathways in hESC-derived hepatic progenitor cells [27]. Here, our data suggest that the ERK pathway is the major signaling pathway by which FGF4 and HGF function during hepatocyte differentiation.

The WNT signaling pathway has been also implicated during the onset of hepatic development, however, the contribution of this signaling is complex [29]. WNT signaling initially inhibits liver induction [34] but shortly afterwards promotes liver bud growth and differentiation [26], [35], [51], [52]. In studies of Xenopus, at early somite stages, WNT signaling acts in the posterior endoderm to repress expression of Hhex, an essential transcriptional regulator of hepatic development. If WNT signaling is blocked in the posterior endoderm, it results in ectopic liver development. Repression of WNT signaling by expression of WNT antagonists in the anterior endoderm is required to relieve repression of Hhex in the anterior endoderm and so facilitate commitment of the endoderm to a hepatic fate. In contract to the repressive effects of WNTs at early somite stages, following specification, WNT signaling appears to promote hepatogenesis in multiple systems including xenopus [35], and Zebrafish [29], [52], [53]. Studies in rodents elucidated the role of wnt1 in directing oval cells (hepatic stem cells in rodents) to differentiate to hepatocytes during the liver regeneration induced by 2-acetylaminofluorene and partial hepatectomy liver regeneration in the rat [30]. Inhibition of WNT1 resulted in failure of hepatocyte differentiation from oval cells, but enhanced cholangiocyte differentiation from oval cells leading to atypical ductular hyperplasia [30]. In our study, WNT1 was significantly down-regulated by ethanol treatment (Figure 2 and 5), and expression of β-catenin and TCF1 was also reduced, suggesting that MAPK/ERK and WNT/β-catenin pathways were synergistically involved in negatively affecting early hepatic differentiation of hESC during EtOH treatment. When we employed the WNT1 inhibitor, the decrease of albumin and TCF1 expression and the reduction of β-catenin in the nucleus occurred as with treatment with ethanol; however, expression of cyclin D1 was not changed (data not shown).

Our data suggest that both ERK and WNT signaling pathways are involved in the hepatic differentiation of hESC after treatment with ethanol. However, the use of the Erk inhibitor appeared to be most effective in replicating the same phenotypic changes as occurred with ethanol treatment. The use of WNT inhibitor resulted in some of the changes shown with ethanol treatment with some differences. For example, use of the WNT inhibitor caused a decrease of albumin, however, the WNT inhibitor did not reduce the expression of cyclin D1. Our assumption is that the Erk pathway was the major pathway regulating hepatocyte differentiation with our culture conditions, and that the WNT pathway was activated and synergistically involved in the hepatocyte differentiation. Ethanol treatment inhibited Erk activation resulting in reduced activation of WNT/β-catenin pathway, which in turn caused the down-regulation of the downstream targets, such as TCF1 and cyclin D1. Another possibility would be that the expression of cyclin D1 can be regulated by the ERK pathway independent of β-catenin. We speculate that these results might explain in part some of the elements of the pathophysiology of alcohol-induced liver disease, for example the inhibition of hepatocyte differentiation and proliferation.

**Figure 6:**
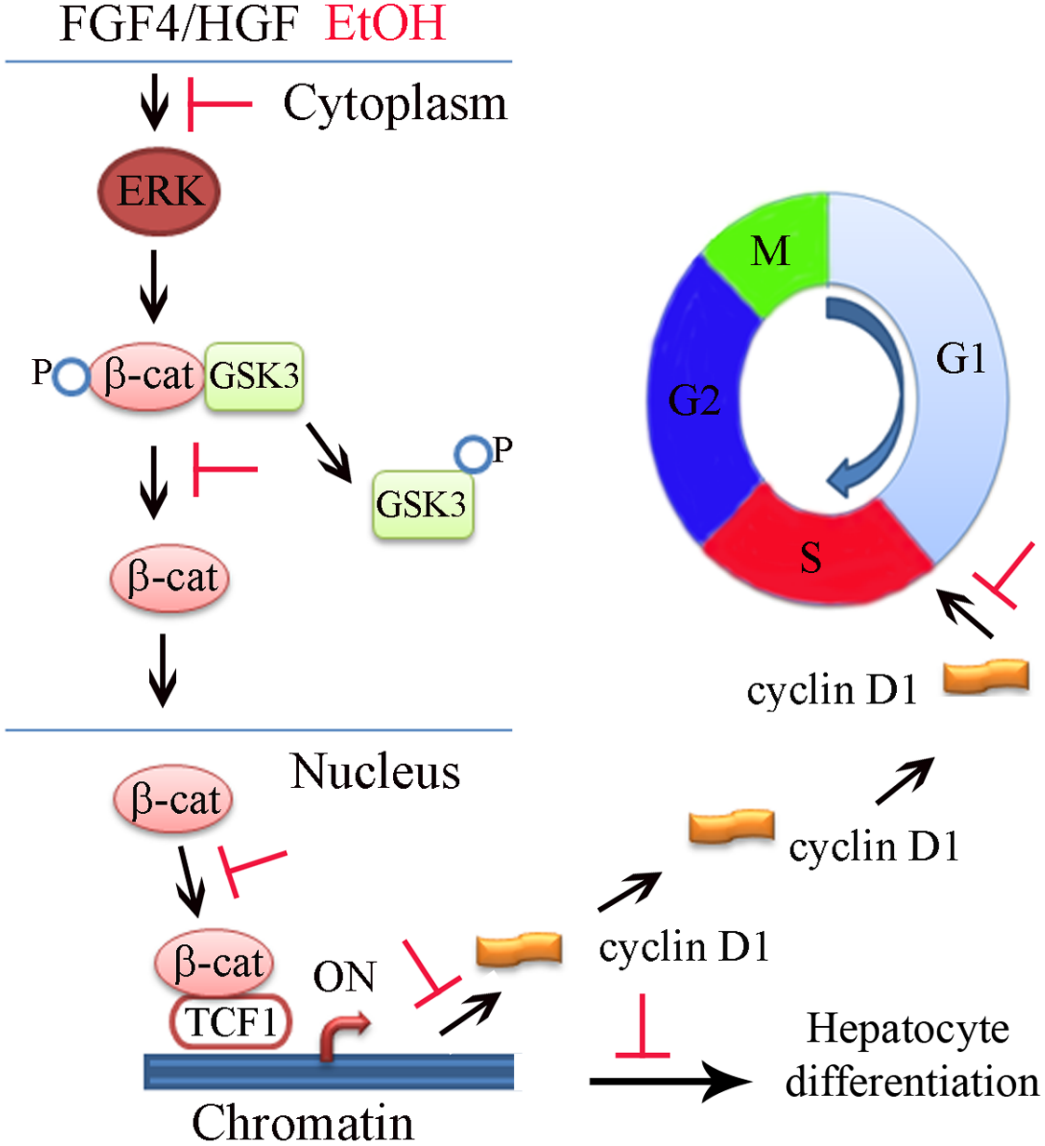
Effects of ethanol on promotion of non-hepatocyte lineage and model for effects of ethanol. Model for effects of ethanol on the differentiation of hESC towards hepatocytes.

In summary, our data demonstrated that ethanol negatively regulate early hepatic differentiation of hESC-derived hepatic progenitor cells, primarily through the inhibition of the MAPK/ERK signaling pathway, and there was also a reduction of cyclin D1 (Figure 6).

## Supporting Information

Figure S1 **Pathway screening by the Signal Transduction PathwayFinder PCR Array.** It was the entire view of the expression changes of 84 genes representing 10 pathways by PCR Array (PAHS-014Z, Sabiosciences) analysis at day 10 after differentiation of human embryonic stem cells in the presence or absence of ethanol at 100 mM. The name of each gene and its expression fold change compared to those in the absence of ethanol is shown in the same spot. The genes and their representing pathway can be found at Sabiosciences website (http://www.sabiosciences.com/rt_pcr_product/HTML/PAHS-014Z.html).
(TIF)

Table S1 **List of antibodies used.** Abbreviations: GAPDH: glyceraldehyde-3-phosphate dehydrogenase.
(DOC)

Table S2 **Information of primers and probes used for qPCR.** Abbreviations: GAPDH, glyceraldehyde-3-phosphate dehydrogenase; AFP, alpha fetoprotein; ASGPR: asialoglycoprotein receptor; α-SMA, alpha smooth muscle actin; C/EBP, CCAAT-enhancer-binding protein.
(DOC)
