# Supplementary figures and images for: Ethanol Negatively Regulates Hepatic Differentiation of hESC by Inhibition of the MAPK/ERK Signaling Pathway In Vitro
Source: PLoS One. 2014 Nov 13;9(11):e112698. doi: 10.1371/journal.pone.0112698 (PMC4231066; doi:10.1371/journal.pone.0112698)

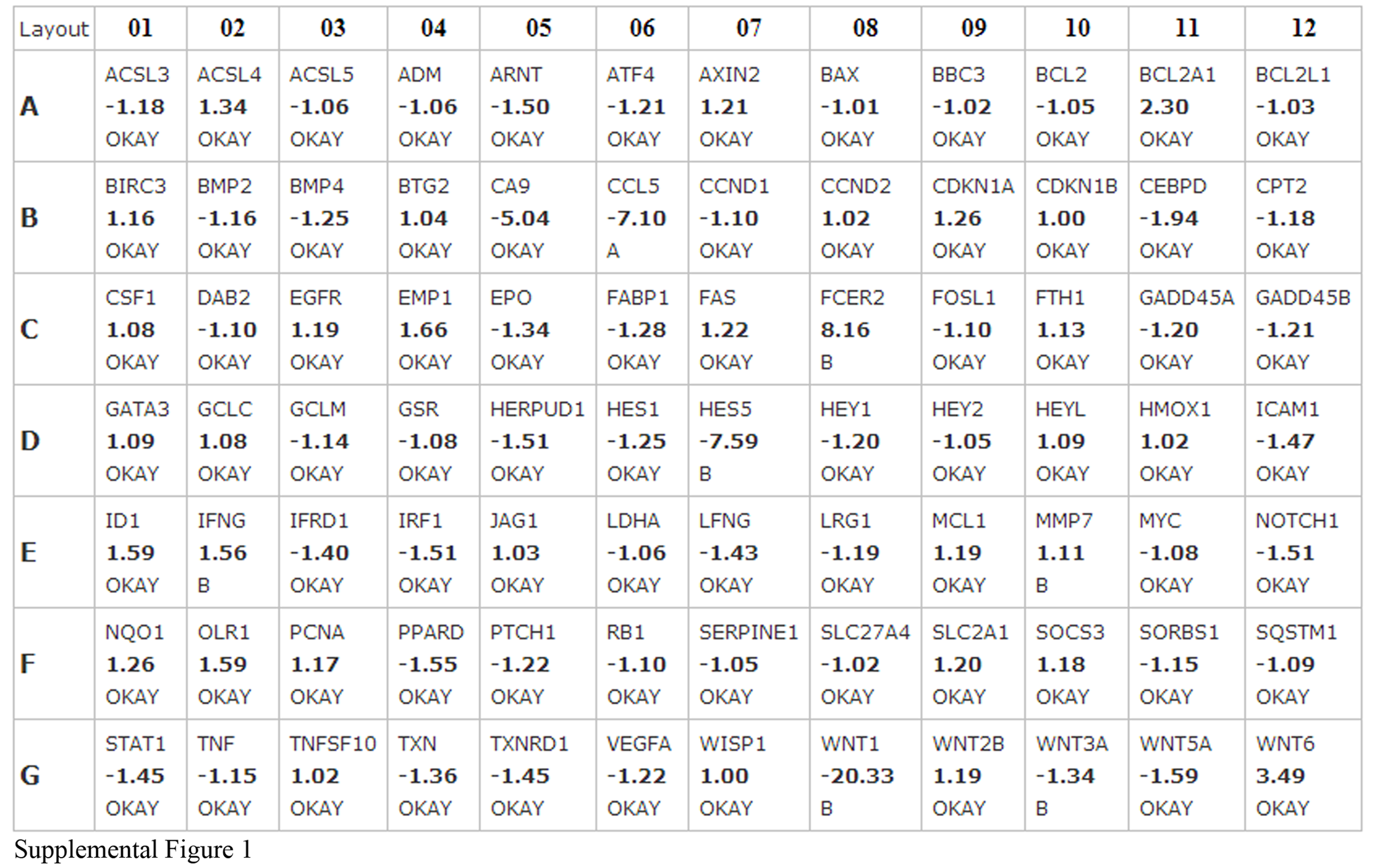

Supplement: Figure S1 — Pathway screening by the Signal Transduction PathwayFinder PCR Array. It was the entire view of the expression changes of 84 genes representing 10 pathways by PCR Array (PAHS-014Z, Sabiosciences) analysis at day 10 after differentiation of human embryonic stem cells in the presence or absence of ethanol at 100 mM. The name of each gene and its expression fold change compared to those in the absence of ethanol is shown in the same spot. The genes and their representing pathway can be found at Sabiosciences website (http://www.sabiosciences.com/rt_pcr_product/HTML/PAHS-014Z.html). (TIF) [file pone.0112698.s001.tif]
