# Supplementary material for: Ethanol Negatively Regulates Hepatic Differentiation of hESC by Inhibition of the MAPK/ERK Signaling Pathway In Vitro
Source: PLoS One. 2014 Nov 13;9(11):e112698. doi: 10.1371/journal.pone.0112698 (PMC4231066; doi:10.1371/journal.pone.0112698)
Supplement: Table S1 — List of antibodies used. Abbreviations: GAPDH: glyceraldehyde-3-phosphate dehydrogenase. (DOC) [file pone.0112698.s002.doc]

Supplemental Table 1. List of antibodies used

| Antigen | Type | Provider | Application |
| --- | --- | --- | --- |
| Albumin | Goat polyclonal | Bethyl | IHC |
| phosphor-AKT | Rabbit polyclonal | Cell Signaling Technology | WB |
| ERK | Rabbit polyclonal | Cell Signaling Technology | WB |
| phosphor-ERK | Rabbit polyclonal | Cell Signaling Technology | WB |
| p38 | Rabbit polyclonal | Cell Signaling Technology | WB |
| phosphor-p38 | Rabbit polyclonal | Cell Signaling Technology | WB |
| JNK | Rabbit polyclonal | Cell Signaling Technology | WB |
| phosphor-JNK | Rabbit polyclonal | Cell Signaling Technology | WB |
| Cyclin D1 | mouse monoclonal | Cell Signaling Technology | WB |
| TCF1 | Rabbit polyclonal | Cell Signaling Technology | WB |
| Wnt1 | Rabbit polyclonal | GeneTex | WB |
| α-Tubulin | Rabbit polyclonal | Cell Signaling Technology | WB |
| β-Catenin | mouse monoclonal | Cell Marque | WB |
| phosphor-GSK3β | Rabbit polyclonal | Cell Signaling Technology | WB |
| GAPDH | mouse monoclonal | Abcam | WB |

Abbreviations: GAPDH: glyceraldehyde-3-phosphate dehydrogenase
