# Supplementary material for: Ethanol Negatively Regulates Hepatic Differentiation of hESC by Inhibition of the MAPK/ERK Signaling Pathway In Vitro
Source: PLoS One. 2014 Nov 13;9(11):e112698. doi: 10.1371/journal.pone.0112698 (PMC4231066; doi:10.1371/journal.pone.0112698)
Supplement: Table S2 — Information of primers and probes used for qPCR. Abbreviations: GAPDH, glyceraldehyde-3-phosphate dehydrogenase; AFP, alpha fetoprotein; ASGPR: asialoglycoprotein receptor; α-SMA, alpha smooth muscle actin; C/EBP, CCAAT-enhancer-binding protein. (DOC) [file pone.0112698.s003.doc]

**Supplemental Table 2. Information of primers and probes used for qPCR**

| Genes | Information or sequences of primers | Application |
| --- | --- | --- |
| Albumin | Hs00609411_m1 (Applied Biosystems) | TaqMan |
| E-cadherin | Hs01013958_m1 | TaqMan |
| GAPDH | Hs99999905_m1 (Applied Biosystems) | TaqMan |
| CK14 | Hs00265033_m1 (Applied Biosystems) | TaqMan |
| N-cadherin | Hs00169953_m1 | TaqMan |
| ASGPR | Hs00155881_m1 | TaqMan |
| AFP | Hs00173490_m1 (Applied Biosystems) | TaqMan |
| CD44 | Hs01075861_m1 (Applied Biosystems) | TaqMan |
| CD146 | Hs00920938_g1 (Applied Biosystems) | TaqMan |
| α-SMA | Hs00426835_m1 (Applied Biosystems) | TaqMan |
| CYP3A4 | Hs00430021_m1 | TaqMan |
| CYP7A1 | Hs00167982_m1 | TaqMan |
| UTG1A6 | Hs01592477_m1 | TaqMan |
| Desmin | Hs00157258_m1 (Applied Biosystems) | TaqMan |
| CD144 | Hs00901463_m1 (Applied Biosystems) | TaqMan |
| HNF1α | Hs01551752_m1 | TaqMan |
| Wnt1 | F:5’- CTGCAGCGACAACATTGACTT-3’  R:5’- GTTGTTGTGAAGGTTCATGAGG-3’ | SYBR |
| Cyclin D1 | F: 5'-GGTCTGCGAGGAACAGAAGTG-3'  R: 5'-TGCAGGCGGCTCTTTTTC-3' | SYBR |
| TCF1 | F: 5’-CCCTACTTTTTATCCCTTGTCTCC-3’  R: 5’-CTGAGGTGTTACAATAGCTGGATG-3’ | SYBR |
| GAPDH | F: 5’-GAAGATGGTGATGGGATTTC-3’  R: 5’-GAAGGTGAAGGTCGGAGTC-3’ | SYBR |
| C/EBP α | F: 5’-CTCGAGGCTTGCCAGACCGT-3’  R: 5’-GCGGGCTTGTCGGGATCTCAG-3’ | SYBR |

Abbreviations: GAPDH, glyceraldehyde-3-phosphate dehydrogenase; AFP, alpha fetoprotein; ASGPR: asialoglycoprotein receptor; α-SMA, alpha smooth muscle actin; C/EBP, CCAAT-enhancer-binding protein.
